# Bone regeneration using moldable calcium phosphate with and without recombinant human BMP-2 in a rabbit critical-sized metaphyseal core defect model

**DOI:** 10.1186/s13018-025-05966-y

**Published:** 2025-06-02

**Authors:** Hyun Seung Ryu, Hyun Jung Park, Mi Young Ryu, Young-Hoon Kim, Sang-Il Kim, Hyung-Youl Park

**Affiliations:** 1 Department of Research Center, CGBio Co., Ltd., Seongnam-Si, Gyeonggi-Do 13211 Republic of Korea; 2 Department of Orthopedic Surgery, Seoul St. Mary’s Hospital, College of Medicine, The Catholic University of Korea, Seoul, 06591 Republic of Korea; 3 Department of Orthopedic Surgery, Eunpyeong St. Mary’s Hospital, College of Medicine, The Catholic University of Korea, 1021, Tongil-ro, Eunpyeong-gu, Seoul, 03321 Republic of Korea

**Keywords:** Bone regeneration, Critical-sized defect, Metaphyseal bone, Moldable calcium phosphate, Recombinant human bone morphogenetic protein-2 (rhBMP-2), Bone graft substitute, Osteoconduction

## Abstract

**Background:**

Moldable calcium phosphate (MCaP) biomaterials have been studied as osteoconductive scaffolds for bone regeneration. However, their potential as carriers for recombinant human bone morphogenetic protein-2 (rhBMP-2) and the biological impact of varying rhBMP-2 doses remain to be fully validated. This study aimed to evaluate the efficacy and safety of MCaP alone and in combination with rhBMP-2 in a rabbit metaphyseal bone defect model.

**Methods:**

Bilateral critical-sized metaphyseal core defects were created in the distal femurs of 73 skeletally mature female New Zealand White rabbits. Animals were assigned to six groups: sham, autograft, MCaP alone, or MCaP combined with low (0.04 mg/cc), mid (0.16 mg/cc), or high (0.6 mg/cc) doses of rhBMP-2. Bone formation and healing were assessed at 3 days and 3, 6, and 12 weeks using radiography, microcomputed tomography (μCT), histomorphometry, and histopathology. Local tissue reactions were evaluated according to ISO 10993–6 standards, and systemic toxicity was assessed through distant organ examinations.

**Results:**

Radiographic and μCT analyses showed progressive bone formation in all treatment groups. Compared with autografts, both the MCaP and rhBMP-2-treated groups exhibited significantly higher bone in the region of interest at 6 and 12 weeks (*p* < 0.05), with no significant differences between the MCaP-only and rhBMP-2 groups. Histological evaluation revealed earlier and more active bone regeneration in rhBMP-2–treated groups, particularly at higher doses. Minimal inflammatory responses were observed across all groups, and no systemic toxicity was detected, supporting the biocompatibility and safety of MCaP-based constructs.

**Conclusions:**

The MCaP carrier demonstrated strong osteoconductive potential and was sufficient to support bone healing compared to autograft in a metaphyseal defect model. The addition of rhBMP-2 promoted earlier bone formation. However, long-term studies in more challenging bone healing environments are warranted to further assess the clinical utility of rhBMP-2 in bone regeneration.

## Background

Certain types of bone defects can be challenging to heal using conventional approaches, especially when the defect is large or structurally complex [1, 2]. Autografts, the current gold standard, provide osteogenic cells, growth factors, and a natural scaffold. However, their clinical use is limited by donor site morbidity, restricted graft volume, and prolonged healing times [3]. Although allografts and synthetic bone substitutes serve as alternatives, they frequently lack the osteoinductive properties necessary for complete bone regeneration [4–6].

Recombinant human bone morphogenetic protein-2 (rhBMP-2) has demonstrated potent osteoinductive potential in both preclinical and clinical studies [7, 8]. By stimulating mesenchymal stem cell differentiation into osteoblasts, rhBMP-2 significantly enhances bone formation [9, 10]. However, clinical application of rhBMP-2 has been challenged by issues such as dose-related complications, short half-life, and the need for localized, sustained delivery at the defect site [11]. Its therapeutic efficacy is therefore highly dependent on the choice of an appropriate carrier [9].

Among various carriers evaluated for rhBMP-2 delivery, absorbable collagen sponges (ACS) have been widely studied and clinically applied. The combination of rhBMP-2 with ACS has been shown to induce bone formation in both preclinical and clinical investigations [12–14]. Notably, INFUSE® Bone Graft (Medtronic Spinal and Biologics, Memphis, TN) was approved by the FDA for specific indications, including spinal fusion and tibial fractures [15]. However, the rapid degradation of ACS may lead to burst release of rhBMP-2, resulting in abnormal bone resorption or heterotopic ossification.

Injectable calcium phosphate matrices (CPMs) have emerged as promising carriers due to their biocompatibility, osteoconductivity, and biodegradability. rhBMP-2 delivered in α-BSM significantly enhanced bone healing in a rabbit ulnar osteotomy model, with superior radiographic union, histological maturity, and mechanical strength, and degradation that mimicked natural bone remodeling [16]. Robust bone formation was also observed in nonhuman primate models using rhBMP-2/CPM, with consistent efficacy across different doses and timing, and reduced transient resorption [17–19]. In a canine delayed-union and primate vertebroplasty model, CPMs demonstrated strong osteoinductive effects and excellent local retention, and the ability to replace PMMA while supporting new bone formation [20, 21]. In a rabbit calvarial defect model, collagen-enhanced biphasic calcium phosphate (BCP) scaffolds promoted early bone formation than other formulations [22]. However, a randomized double-blind trial in patients with closed tibial fractures reported no significant improvement in healing or pain-free weight-bearing, likely due to poor retention and uncontrolled diffusion of the injectable material [23].

Moldable calcium phosphate (MCaP) has recently gained attention as a next-generation carrier. In particularly, MCaP improves upon traditional CaP carriers by increasing solid content to reduce rapid diffusion, adapting to irregular defect geometries, and enabling controlled release of bioactive molecules, making it an ideal platform for rhBMP-2 delivery [24]. Nevertheless, systematic investigations comparing rhBMP-2 concentrations within a defined MCaP matrix and exploring the interaction between carrier resorption dynamics and bone formation remain limited [25–28].

Thus, the present study aims to evaluate the osteoconductive potential of an MCaP carrier and the supplementary effect of rhBMP-2 using a critical-sized metaphyseal core defect model in the distal femoral condyle of rabbits [29–31]. The findings are expected to clarify the carrier’s suitability in trabecular bone environments and assess whether the addition of rhBMP-2 provides further benefit in bone healing conditions.

## Methods

### Animal Model

A total of 73 skeletally mature female New Zealand White rabbits were randomly assigned to six experimental groups (Table 1). Each group consisted of 24 animals, further subdivided into sets of eight for radiographic and histological assessments at predetermined time points (3 days and 3, 6, and 12 weeks). These assessments evaluated bone formation, maturation, and potential adverse healing events. All procedures adhered to ethical guidelines and were approved by the Animal Care and Ethics Committee (Approval No.: UNSW 20/144A).

**Table 1:** Study design and observation timeline

Observation interval	Sham	Autograft	MCaP carrier control	MCaP carrier with rhBMP-2		
				Low-dose (0.04 mg/cc)	Mid-dose (0.16 mg/cc)	High-dose (0.6 mg/cc)
	*N* = 16 (8)	*N* = 26 (13)	*N* = 26 (13)	*N* = 26 (13)	*N* = 26 (13)	*N* = 26 (13)
3 days	NA	2 (1)	2 (1)	2 (1)	2 (1)	2 (1)
3 weeks	NA	8 (4)	8 (4)	8 (4)	8 (4)	8 (4)
6 weeks	8 (4)	8 (4)	8 (4)	8 (4)	8 (4)	8 (4)
12 weeks	8 (4)	8 (4)	8 (4)	8 (4)	8 (4)	8 (4)

*NA *Not applicable

Numbers in parentheses indicate the number of animals

### Preparation of Graft Materials

Six experimental groups were established: Sham, Autograft, MCaP carrier control, and three treatment groups with low (0.04 mg/cc), mid (0.08 mg/cc), and high (0.6 mg/cc) rhBMP-2.

The positive control group (iliac crest bone graft) received autografts harvested from the iliac crest, which were finely minced and prepared for implantation. For other groups treated with MCaP, pre-packaged graft material kits (NOVOSIS™ Putty, CGbio Co., Ltd., Seongnam, Korea) were used. Each kit contained hydroxyapatite (HA), β-tricalcium phosphate (β-TCP)/hydrogel, lyophilized rhBMP-2, and sterile water for injection.

**Fig. 1:**
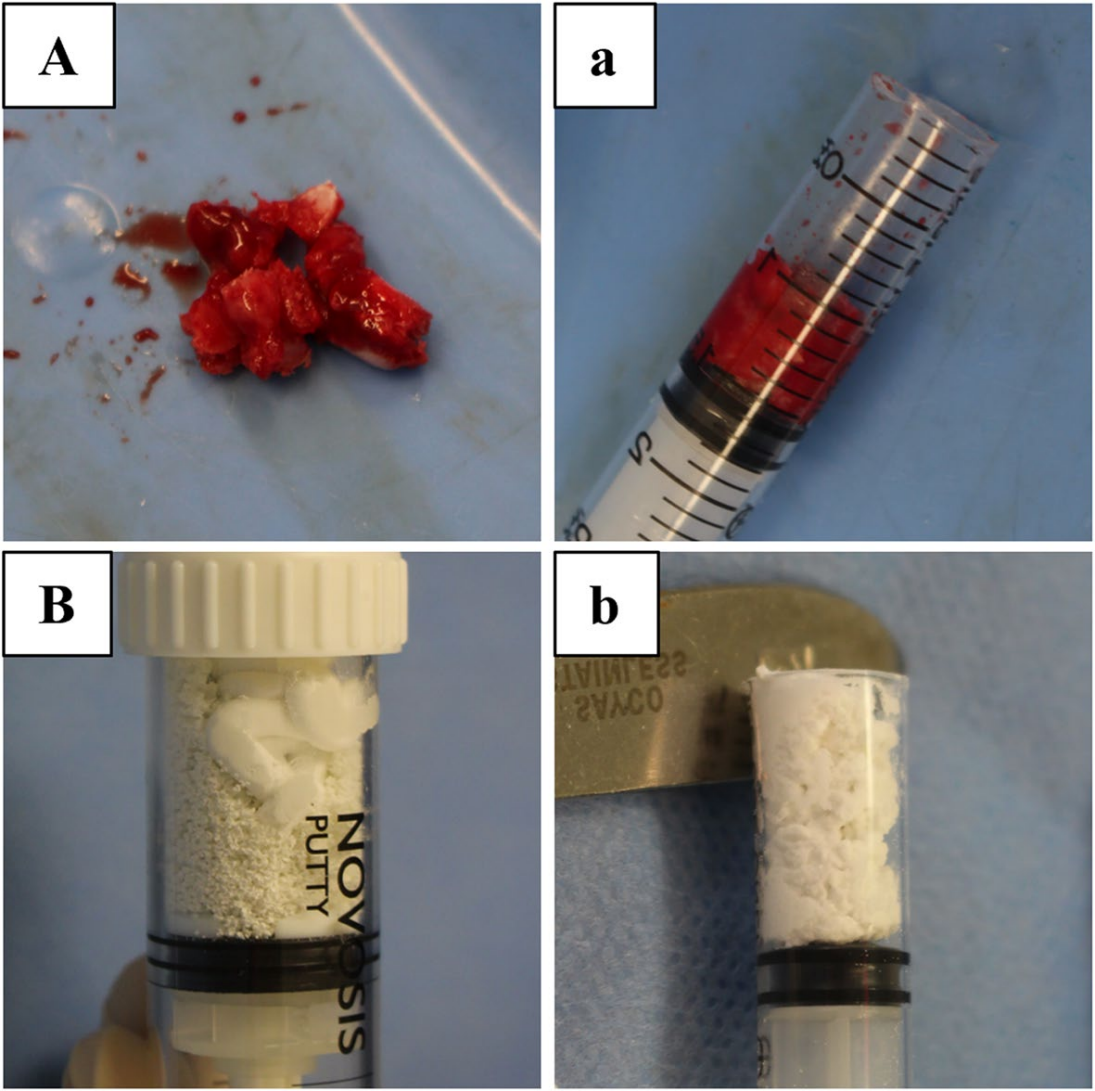
Preparation of graft materials (**A**) Iliac crest bone graft (ICBG) harvested from a rabbit, (**a**) finely chopped, and loaded into a syringe. **B** MCaP carrier materials before and (**b**) after mixing

The graft materials were prepared immediately before implantation using a standardized mixing protocol. For rhBMP-2-treated groups, the lyophilized rhBMP-2 was first reconstituted with sterile water and then absorbed into HA granules. This mixture was subsequently combined with the β-TCP/hydrogel component to form a cohesive putty. For groups not receiving rhBMP-2, the HA granules were hydrated using sterile water alone, following the same mixing steps to maintain consistency in material handling [10–12]. Each rhBMP-2 group received the designated concentration (0.04, 0.16, or 0.6 mg/cc), and all formulations were prepared in identical volumes and consistency across groups. A detailed description of the graft material is shown in Fig. 1.

### Surgical Procedures

**Fig. 2:**
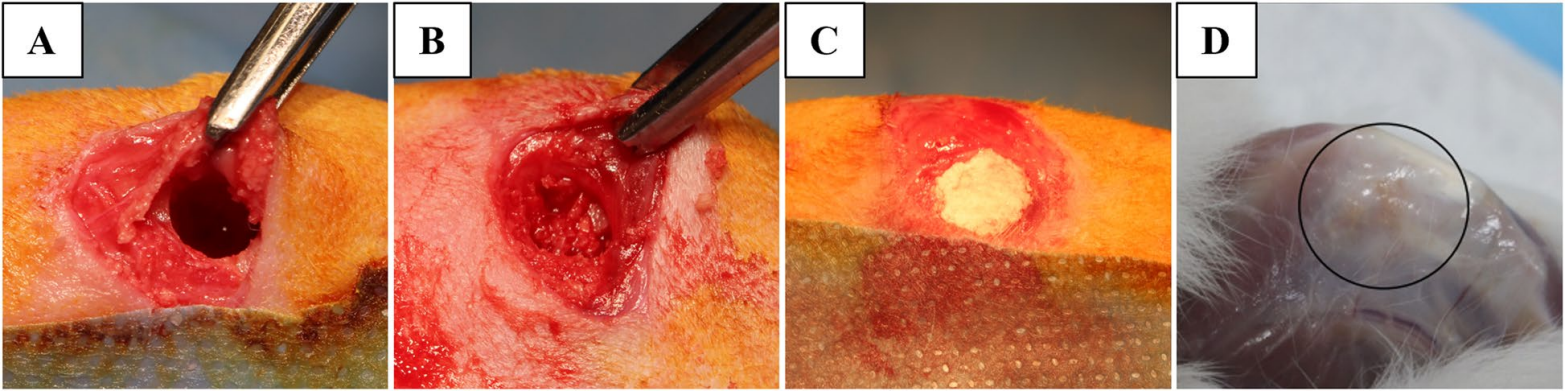
Critical-sized defect creation and graft application (**A**) Sham group – defect left unfilled, **B** Iliac crest bone graft, **C** MCaP carrier, applied alone and with three different rhBMP-2 dosages, **D** Postoperative surgical site following specimen harvest

Bilateral critical-sized defects (6 mm in diameter and 10 mm in depth) were created in the cancellous bone of the distal femur. The defects were initially drilled with a 4.5-mm drill, followed by a 6-mm drill, and then refined using a flat-end mill under continuous saline irrigation. Each defect was filled with approximately 0.3 cc of the designated graft material using a spatula. Digital photographs were taken to document the surgical procedure (Fig. 2). The skin was closed with 3–0 Dexon sutures, and postoperative analgesia (Temgesic, 1 ml, subcutaneously) was administered. Animals were allowed to mobilize and bear weight as tolerated.

Postoperative monitoring was conducted daily until the study endpoint. Observation sheets were recorded daily for the first 7 days and weekly thereafter. Cage-side assessments included general health parameters, such as skin and fur condition, ocular and mucosal integrity, and respiratory, circulatory, autonomic, and central nervous system function. Behavioral observations were also documented.

### Sacrifice and tissue harvest

At designated time points, the animals were weighed, scanned to confirm microchip identification, and anesthetized with isoflurane inhalation. Euthanasia was performed through a lethal intracardiac injection of pentobarbitone sodium. While under anesthesia, the right and left femora were harvested and photographed. Internal organs, including the heart, liver, kidney, lungs, and lymph nodes, were inspected, photographed, weighed, and processed for routine paraffin histology. The veterinary pathologist reviewed distant organs for any abnormalities.

### Radiography and Microcomputed Tomography (μCT)

Following tissue harvesting, the femora were radiographed using a Faxitron system (Faxitron Bioptics, LLC, Tucson, AZ, USA) with mammography film and digital plates (24 kV, 45 s) on both anteroposterior (AP) and lateral views. Images were digitally processed, and DICOM data were converted into JPG format for analysis.

Microcomputed tomography (μCT) imaging was performed on all samples using an Inveon in vivo scanner (Siemens Medical Solutions, Knoxville, TN, USA) to obtain high-resolution images of the implantation site. Raw images were reconstructed to DICOM format and analyzed in axial, sagittal, and coronal planes to assess bone healing and adverse events [32]. Image acquisition was performed at a slice thickness of 20 microns, and 3D models of the implantation site were generated for each animal.

### Tissue processing and histomorphometry

All distal femur specimens were fixed in 10% phosphate-buffered formalin for at least 96 h before embedding in either polymethyl methacrylate (PMMA) or paraffin. PMMA Sects. (15 μm) were stained with methylene blue and basic fuchsin, while paraffin-embedded sections were decalcified, sectioned, and stained with hematoxylin and eosin (H&E).

Histomorphometric analysis was conducted on PMMA-stained sections. A validated custom MATLAB program (Version 3.3) was used to quantify the area percentages of graft material, bone tissue (including new bone and marrow), and soft tissue [29, 33–35]. Three low-magnification images (1.25 × magnification, 1 mm scale bar) were captured from each of the three PMMA slides per animal.

Regions of interest (ROIs) were identified and delineated using a polygon selection technique. Tissue types, including graft material, mineralized bone, bone marrow, and fibrous tissue, were classified based on pixel color and morphology. The area percentage of each tissue type within the ROI was calculated, and a mean value was determined for each animal [36]. Bone in the region of interest (BIRI) was quantified as Bone/(1—Material) over time.

### Histopathology

Paraffin-embedded sections were examined under light microscopy with an Olympus DP72 camera by a blinded pathologist. Key histological parameters, including residual test material, new bone formation, and inflammatory responses, were assessed at magnifications ranging from 4 × to 40 × . Grading was conducted following ISO 10993–6 biological evaluation standards using a carrier control as the predicate. Evaluation criteria were: minimal or no reaction (0.0–2.9), slight (3.0–8.9), moderate (9.0–15.0), and severe (≥ 15.1) [37].

### Statistical analysis

Continuous variables were presented as means and standard deviations (SD), with between-group differences analyzed using the Student’s t-test or Mann–Whitney U test. For multiple group comparisons, an analysis of variance (ANOVA) was conducted, followed by a Games-Howell post-hoc test. All statistical analyses were performed using SPSS software (IBM SPSS Statistics, Version 26.0), with a *p*-value < 0.05 considered statistically significant.

## Results

### Clinical observations

All animals were confirmed to be clinically healthy before enrollment and remained so throughout the study. Skeletal maturity was verified radiographically by confirming growth plate closure. Surgery was successfully performed on 73 animals without intraoperative complications, and all animals recovered uneventfully.

Blood biochemistry, hematology, and distant organ histology were assessed by the veterinary pathologist. No adverse findings or systemic reactions were observed in animals sacrificed at 3 days, and at 3, 6, and 12 weeks postoperatively, compared with preoperative samples.

### Radiography

Faxitron radiographs confirmed consistent defect placement throughout the study. Direct comparisons between groups were challenging due to the presence of adjacent normal bone. The negative control (empty defect) and positive control (autograft) exhibited periosteal reactions consistent with surgical trauma.

**Fig. 3:**
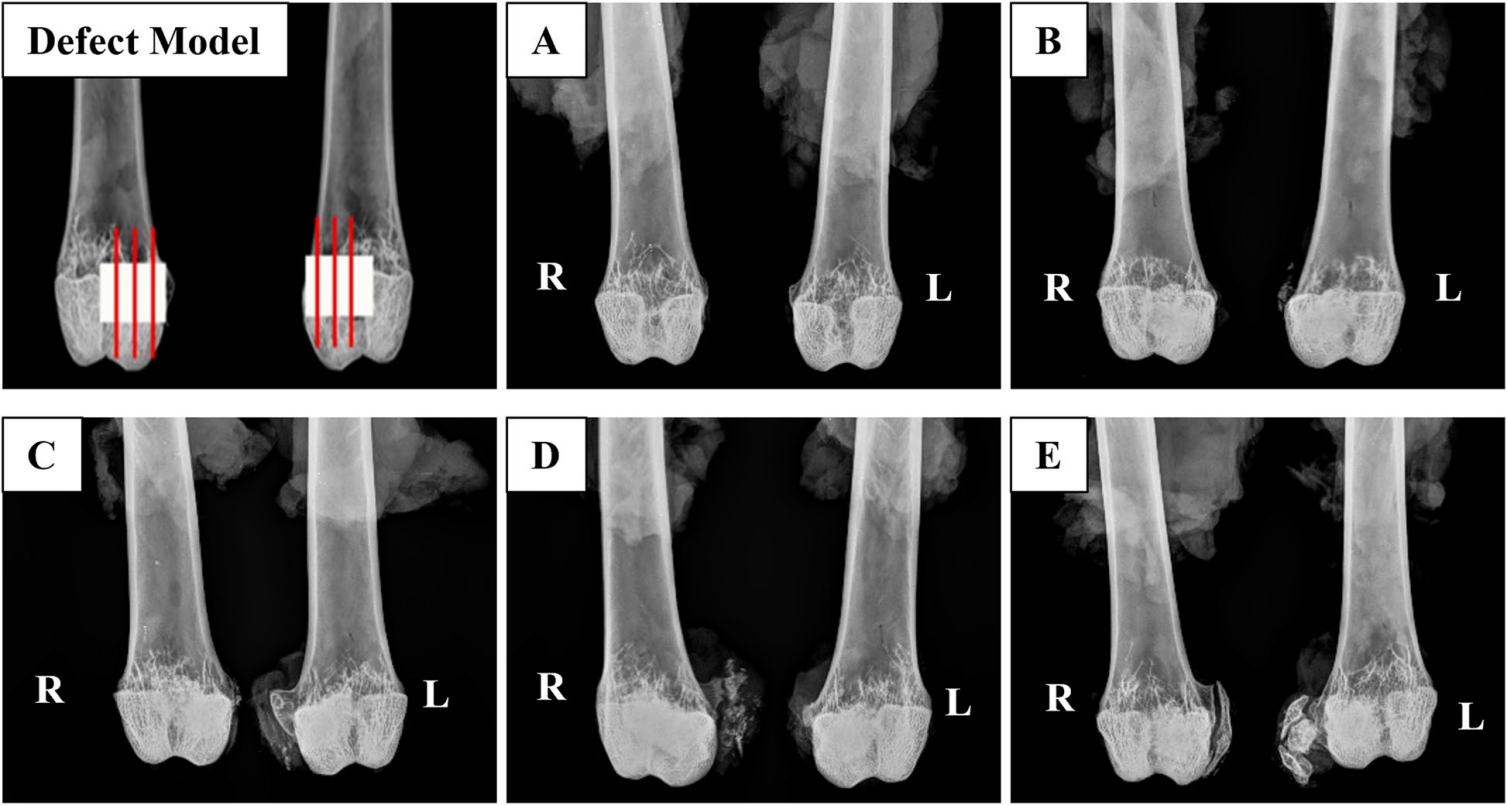
Representative Faxitron radiographs at 12 weeks. **A** Left: Empty defect, Right: Autograft, **B** Left and Right: MCaP carrier control, **C** Left and Right: MCaP carrier with rhBMP-2 (0.04 mg/cc), **D** Left and Right: MCaP carrier with rhBMP-2 (0.16 mg/cc), **E** Left and Right: MCaP carrier with rhBMP-2 (0.6 mg/cc)

In the MCaP control and rhBMP-2 treatment groups (MCaP with rhBMP-2 at 0.04 mg/cc, 0.16 mg/cc, and 0.6 mg/cc), graft material remained visible at all time points. Additionally, ectopic bone formation outside the defect areas was observed (Fig. 3).

### Micro-CT Analysis

**Fig. 4:**
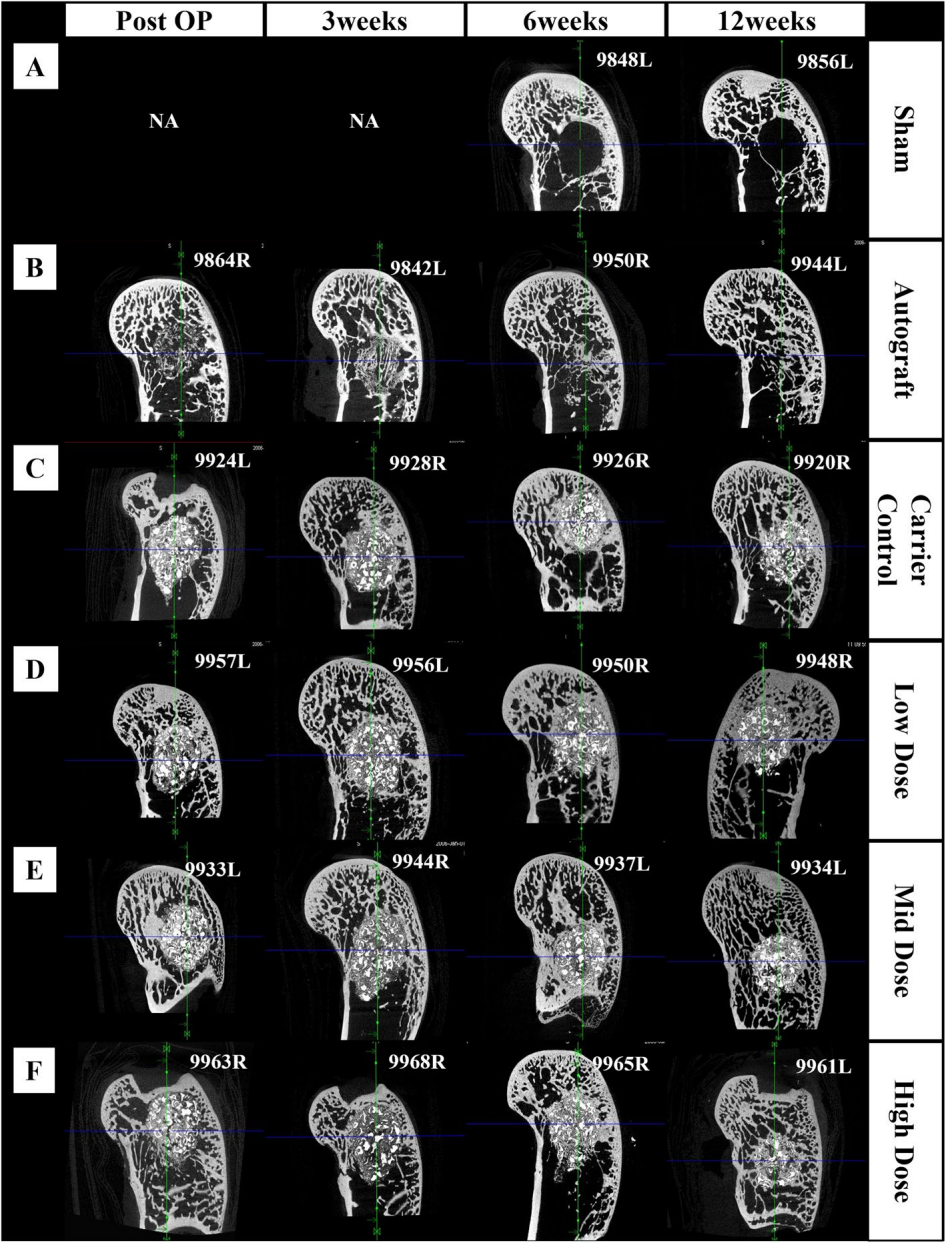
Representative μCT images at 0, 3, 6, and 12 weeks post-implantation. **A** Sham (empty defect), **B** Autograft, **C** MCaP carrier control, **D** MCaP carrier with rhBMP-2 (0.04 mg/cc, low dose), **E** MCaP carrier with rhBMP-2 (0.16 mg/cc, mid dose), **F** MCaP carrier with rhBMP-2 (0.6 mg/cc, high dose)

μCT confirmed consistent defect positioning across all groups. Figure 4 presents representative μCT images at multiple time points post-implantation. Minimal bone formation was observed in the sham group up to 12 weeks, confirming the critical nature of the defect model. In contrast, defects treated with autografts demonstrated progressive healing, characterized by bone remodeling over time. The MCaP control and all rhBMP-2-treated groups exhibited gradual new bone formation throughout the study period. Progressive resorption of calcium phosphate was observed in both the MCaP control and rhBMP-2 treatment groups. However, complete resorption was not achieved in any group by the 12-week endpoint.

### Histomorphometry

Histologic analysis of PMMA-embedded sections was used to quantify the mean percentages of bone, material, marrow, and other tissue (e.g., fibrous tissue) within each treated defect. BIRI was also calculated. Table 2 summarizes the histomorphometric data.

**Table 2:** Summary of histomorphometry data

Group	Weeks	Bone (± SD)	Material (± SD)	Marrow (± SD)	Other (± SD)	BIRI		
						**(± SD)**	*P***-value** **(vs Autograft)**	*P***-value** **(vs Carrier)**
**Sham**	6	0.06 (± 0.04)	0.0	0.93 (± 0.05)	0.01 (± 0.01)	0.06 (± 0.04)	0.001	< 0.001
	12	0.07 (± 0.04)	0.0	0.93 (± 0.04)	0.0	0.07 (± 0.04)	0.077	< 0.001
**Autograft**	0.4	0.53 (± 0.0)	0.0	0.0	0.47 (± 0.0)	0.53 (± 0.0)	-	-
	3	0.54 (± 0.09)	0.0	0.39 (± 0.17)	0.07 (± 0.12)	0.54 (± 0.09)	-	0.424
	6	0.53 (± 0.09)	0.0	0.43 (± 0.12)	0.04 (± 0.03)	0.53 (± 0.09)	-	0.002
	12	0.37 (± 0.13)	0.0	0.63 (± 0.13)	0.0	0.37 (± 0.13)	-	0.035
**Carrier Control**	0.4	0	0.79 (± 0.0)	0.0 (± 0.0)	0.21 (± 0.0)	0.0	-	-
	3	0.28 (± 0.12)	0.64 (± 0.08)	0.01 (± 0.01)	0.07 (± 0.08)	0.76 (± 0.21)	0.424	-
	6	0.47 (± 0.05)	0.49 (± 0.05)	0.02 (± 0.02)	0.01 (± 0.01)	0.94 (± 0.03)	0.002	-
	12	0.43 (± 0.04)	0.44 (± 0.06)	0.09 (± 0.02)	0.04 (± 0.03)	0.77 (± 0.03)	0.035	-
**Low-dose** **(0.04 mg/cc)**	0.4	0	0.63 (± 0.0)	0	0.37 (± 0.0)	0	-	-
	3	0.39 (± 0.02)	0.49 (± 0.05	0.08 (± 0.07)	0.05 (± 0.03)	0.76 (± 0.09)	0.119	1.000
	6	0.47 (± 0.08)	0.46 (± 0.07)	0.06 (± 0.04)	0.01 (± 0.01)	0.86 (± 0.08)	0.006	0.611
	12	0.46 (± 0.06)	0.39 (± 0.06)	0.14 (± 0.08)	0.01 (± 0.01)	0.76 (± 0.11)	0.029	1.000
**Mid-dose** **(0.16 mg/cc)**	0.4	0	0.71 (± 0.0)	0	0.29 (± 0.0)	0	-	-
	3	0.43 (± 0.11)	0.47 (± 0.09	0.09 (± 0.04)	0.02 ± 0.02	0.8 (± 0.11)	0.052	0.993
	6	0.4 (± 0.03)	0.52 (± 0.06)	0.07 (± 0.03)	0.01 ± 0.01	0.85 (± 0.04)	0.005	0.058
	12	0.44 (± 0.03)	0.39 ± 0.03	0.15 (± 0.04)	0.01 (± 0.02)	0.73 (± 0.07)	0.034	0.877
**High-dose** **(0.6 mg/cc)**	0.4	0	0.7 (± 0.0)	0	0.31 (± 0.0)	0.0	-	-
	3	0.39 (± 0.05)	0.51 (± 0.03)	0.03 (± 0.01)	0.07 (± 0.03)	0.8 (± 0.07)	0.026	0.993
	6	0.49 (± 0.06)	0.4 (± 0.07)	0.11 (± 0.06)	0	0.82 (± 0.09)	0.014	0.304
	12	0.47 (± 0.05)	0.36 (± 0.08)	0.16 (± 0.13)	0.01 (± 0.01)	0.75 (± 0.15)	0.062	1.000

Bone in the region of interest (BIRI) = Bone/(1—Material) over time

**Fig. 5:**
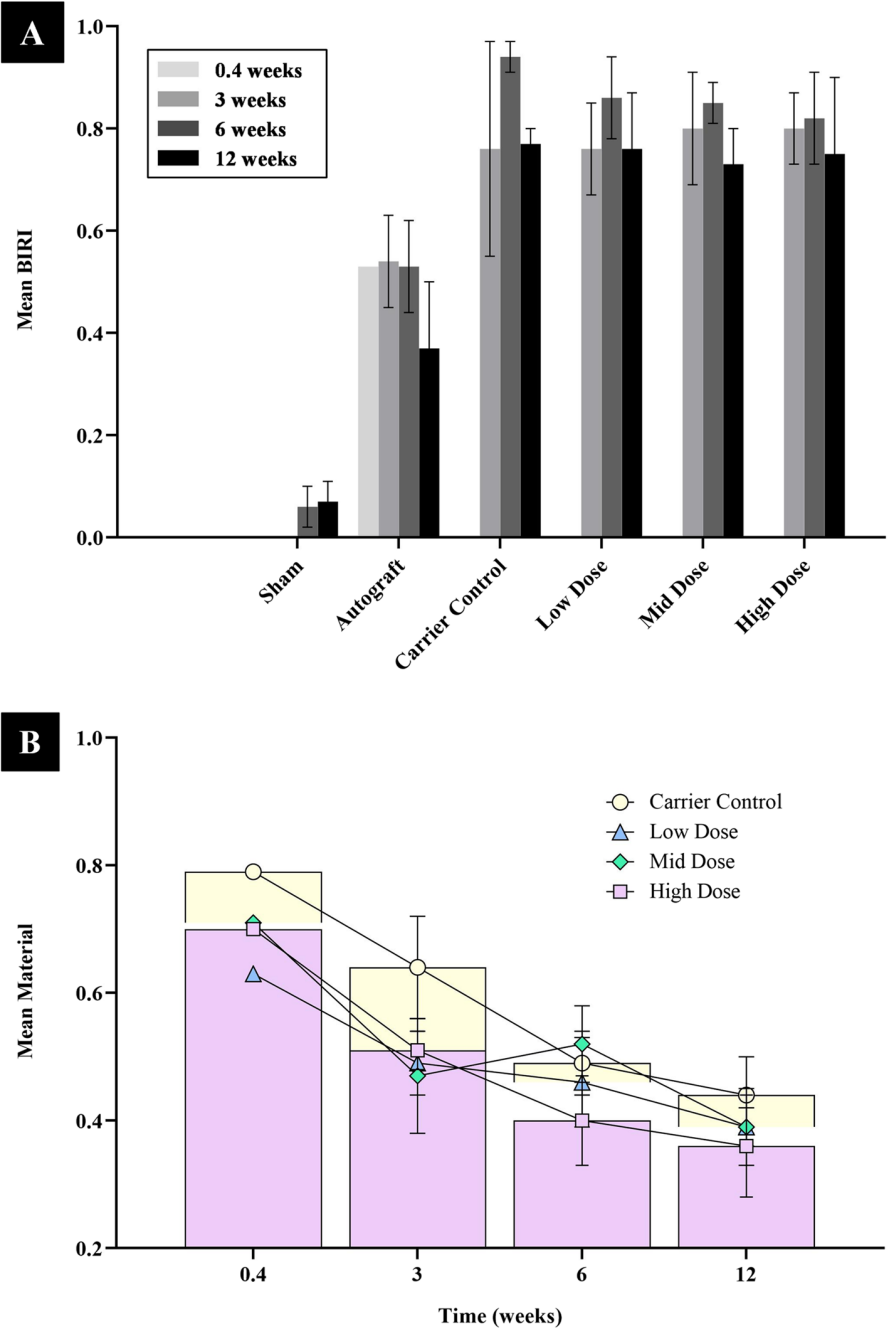
Histomorphometric analysis across six groups at 3 days and 3, 6, and 12 weeks post-implantation. **A** Bone in the region of interest (BIRI), calculated as Bone/(1 – Material). **B** Residual material graph showing the percentage of remaining graft material over time. Line graphs represent all groups; bar graphs in yellow (Carrier Control) and pink (High Dose) highlight group differences at selected time points

The mean bone percentage in the autograft group was comparable to that in the MCaP carrier and rhBMP-2 groups at 3, 6, and 12 weeks (all *p* > 0.05). However, the BIRI exhibited significant differences over time (Fig. 5A).

At 3 weeks, only the high-dose rhBMP-2 group demonstrated a significantly higher BIRI compared to the autograft group (0.8 ± 0.07 vs. 0.54 ± 0.09, *p* = 0.026). By 6 weeks, the MCaP carrier and all rhBMP-2-treated groups exhibited significantly higher BIRI values compared to the autograft group (all *p* < 0.05). At 12 weeks, BIRI in the autograft remained significantly lower than in the carrier group (*p* = 0.035) and the low- and mid-dose rhBMP-2 groups (*p* = 0.029, *p* = 0.034), while the high-dose group showed a trend toward significance (*p* = 0.062). However, there were no significant differences in BIRI values between the MCaP carrier group and the rhBMP-2–treated groups at 3, 6, and 12 weeks (all *p* > 0.05).

Both the MCaP carrier control and rhBMP-2-treated groups (low, mid, and high-dose) demonstrated progressive resorption of carrier materials over 12 weeks. Resorption occurred at a slightly faster rate in groups receiving higher rhBMP-2 doses (Fig. 5B).

### Histopathology

Paraffin histology images revealed minimal healing in the sham group (negative control), with defects largely occupied by fibrous and fatty marrow tissue. New bone formation remained below 10% through 12 weeks, confirming the critical nature of the defect model. In contrast, autograft-treated defects (positive control) showed continuous healing, characterized by new woven bone formation followed by remodeling.

The MCaP carrier-only group demonstrated osteoconductive properties. As spherical β-TCP and HA gradually resorbed, prominent osteogenic cell attachment was observed around β-TCP and HA, indicating active bone formation and remodeling by 6 weeks. Compared to the carrier control, all rhBMP-2-treated groups (0.04, 0.16, or 0.6 mg/cc) exhibited increased bone formation, with earlier and more active healing, beginning as early as 3 weeks. In these group, faster resorption of β-TCP and HA was observed, and bone formation continued throughout the 12 weeks. Higher rhBMP-2 doses were associated with more rapid and extensive osteogenesis.

**Fig. 6:**
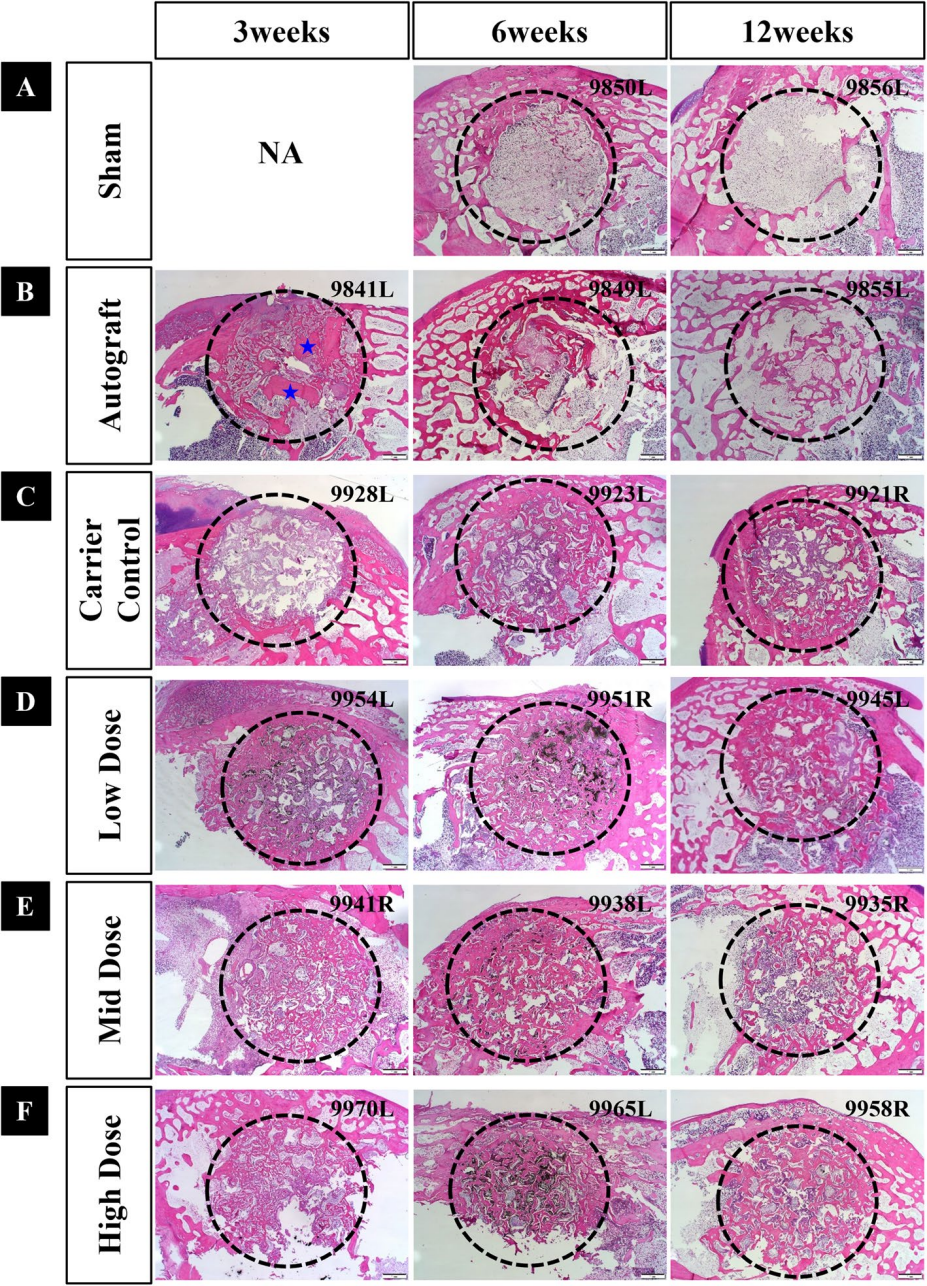
Representative low-magnification paraffin histological images (scale bar = 1 mm) Histological images of the six groups at 3, 6, and 12 weeks post-implantation. **A** Sham (empty defect), **B** Autograft, **C** Carrier control, **D** Low dose (0.04 mg/cc BMP), **E** Mid dose (0.16 mg/cc BMP), and **F** High dose (0.6 mg/cc BMP). The defect and implantation sites are outlined in circles. Symbol in histology image: Blue star (★), residual autograft material

**Fig. 7:**
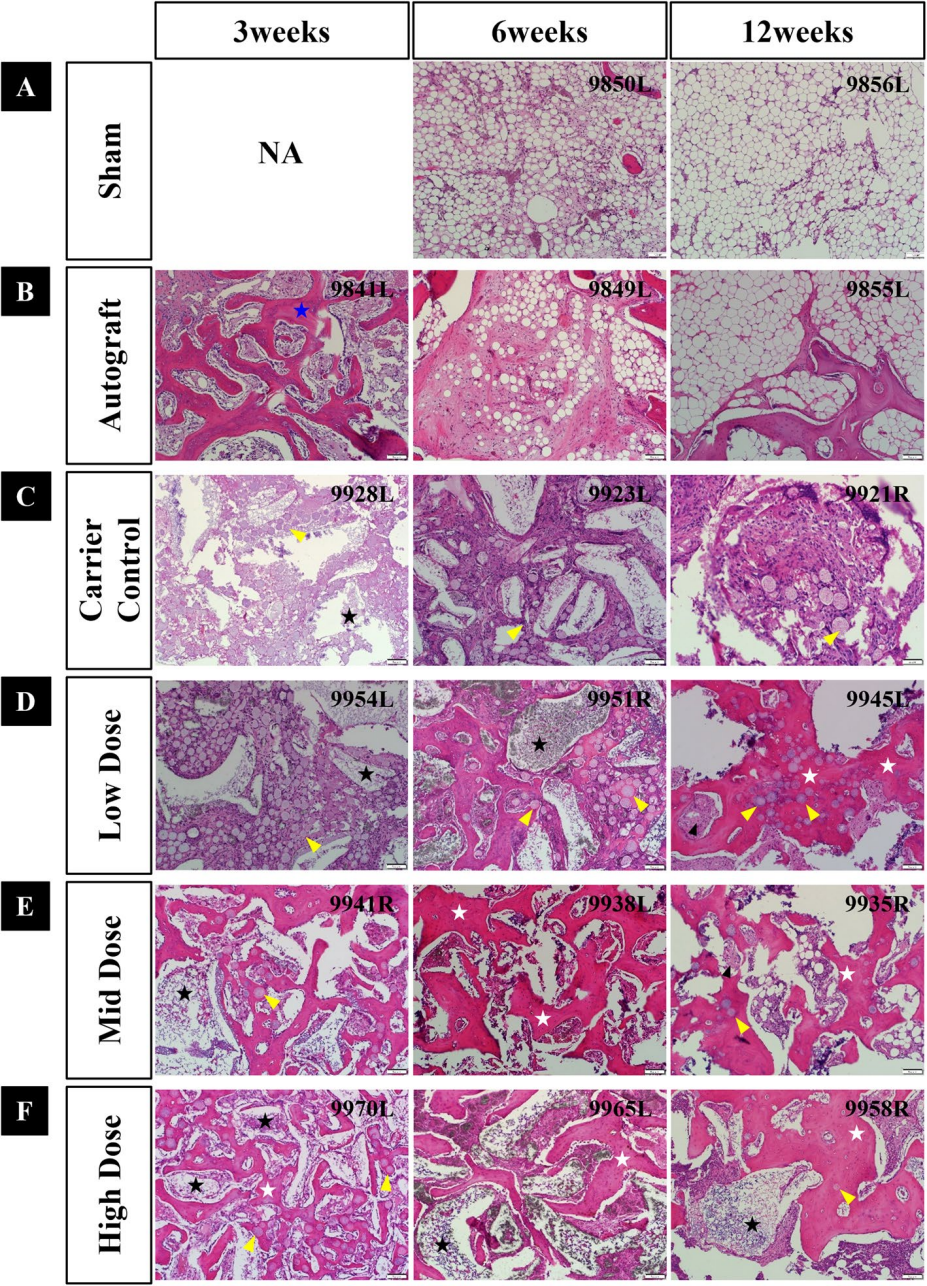
Representative high-magnification paraffin histological images (scale bar = 100 microns). Histologic images of the six groups at 3, 6, and 12 weeks post-implantation. **A** Sham (empty defect), **B** Autograft, **C** Carrier control, **D** Low dose (0.04 mg/cc), **E** Mid dose (0.16 mg/cc), and **F** High dose (0.6 mg/cc). Symbol in histology image: Black star (★), HA granules; Blue star (★), residual autograft material; White star (▮), new bone formation; Yellow triangle (  ), β-TCP beads; Black triangle (  ), marrow development/formation

Representative paraffin histology images at different time points are shown in Fig. 6 (low magnification, × 1.25) and Fig. 7 (high magnification, × 10).

### Quantitative evaluation of local tissue reactions

The quantitative evaluation of local tissue reactions, assessed in accordance with ISO 10993–6, is summarized in Table 3. Across all time points, all test groups exhibited minimal to no reaction (scores ranging from −5.13 to 2.83), confirming the biocompatibility of the graft materials combined with rhBMP-2.

**Table 3:** Tissue irritation scoring results

Groups Time points	Low-dose (0.04 mg/cc)	Mid-dose (0.16 mg/cc)	High-dose (0.6 mg/cc)
3 weeks	2.08	2.50	0.67
6 weeks	0.67	0.58	1.22
12 weeks	−2.63	2.83	−5.13

Carrier control serves as the reference group

## Discussion

MCaP biomaterials offer several clinical advantages, particularly in bone defect healing [27, 38]. Their osteoconductive properties facilitate bone ingrowth and remodeling, while their moldability ensures precise adaptation to defect morphology, promoting cellular infiltration and integration with host bone [39, 40]. Unlike particulate grafts, which are prone to migration, MCaP provides a stable scaffold that maintains structural integrity throughout the healing process. Additionally, the gradual resorption of MCaP allows for progressive replacement by newly formed bone, supporting the remodeling process necessary for successful defect healing [41–43].

In this study, the MCaP carrier consistently remained within the defect site over the 12-week period, gradually resorbing and being replaced by newly formed bone. Radiographic assessments confirmed this remodeling process, demonstrating increased mineralization and progressive integration with host bone. These results emphasize the importance of scaffold stability in promoting consistent bone regeneration and minimizing complications associated with material migration [44, 45].

Histomorphometric analysis demonstrated that the MCaP carrier effectively supported bone formation. The MCaP group exhibited higher BIRI values than the autograft group at 6 and 12 weeks, suggesting favorable osteoconductive properties [27, 38]. At 3 weeks, bone formation was comparable across all groups, but the high-dose rhBMP-2 group showed significantly greater BIRI than the autograft. While the rhBMP-2–treated groups showed a trend toward increased bone formation at later time points, the differences in BIRI values were not statistically significant when compared to the MCaP carrier group.

Histopathologic analysis confirmed expected remodeling and woven bone formation in the autograft group. The MCaP carrier also supported active bone formation by 6 weeks, consistent with its inherent osteoconductivity. In the rhBMP-2–treated groups, particularly at higher doses, more rapid resorption of β-TCP and HA granules was observed, along with earlier and more active bone healing compared to the carrier-only group. These findings are consistent with the known biological effects of rhBMP-2, which promotes bone regeneration by recruiting mesenchymal stem cells and inducing their differentiation into osteoblasts [46, 47]. The qualitative histological findings suggest that rhBMP-2 may enhance the early phases of bone healing, even in biologically favorable metaphyseal bone defects [48].

Interestingly, while histomorphometric analysis based on BIRI did not show statistically significant differences between the MCaP carrier and BMP-treated groups, histological assessment revealed more advanced bone bridging and marrow cavity remodeling in the high-dose BMP group. This discrepancy may be attributed to the highly vascular, trabecular nature of metaphyseal bone, which may promote bone formation uniformly across groups regardless of BMP administration [49].

Furthermore, ectopic bone formation was observed in the rhBMP-2–treated group, particularly at higher doses. This may have resulted from early release or diffusion of rhBMP-2 beyond the intended defect site, facilitated by the porous, well-perfused nature of metaphyseal bone as well as the relatively rapid degradation of poloxamer hydrogel within the carrier. While ectopic ossification is a known concern in BMP-based therapies, it also reflects the strong osteoinductive potential of rhBMP-2. When properly contained and controlled, this may accelerate bone regeneration in clinically relevant settings [50].

These findings suggest that carrier-only systems such as MCaP may be sufficient for bone healing in favorable metaphyseal environments. However, rhBMP-2 retains considerable value, especially in accelerating early healing phases [17, 51]. The previous studies have reported that the benefits of rhBMP-2 may be more evident in diaphyseal segmental defects or other environments with limited osteogenic potential, where natural healing is insufficient and additional osteoinductive support is needed [52–54].

Safety assessment revealed no significant inflammatory or adverse tissue responses in any experimental groups, including those treated with rhBMP-2. Evaluation on ISO 10993–6 indicated minimal to no adverse tissue responses, reinforcing the biocompatibility of rhBMP-2 in combination with MCaP for clinical use [37]. These findings suggest that the controlled release of rhBMP-2 from MCaP may mitigate the complications typically associated with high-dose BMP-2 administration, such as excessive bone resorption or inflammatory reactions [44, 55].

This study has several limitations. First, the 12-week observation period limited the assessment of long-term scaffold resorption and bone remodeling. Future studies should include extended observation periods to evaluate sustained bone regeneration, scaffold integration, and potential late-stage remodeling effects [56]. Second, while the rabbit femoral condyle model is widely accepted for preclinical bone regeneration research, it primarily represents metaphyseal trabecular bone, which has a favorable biological environment for healing [57]. This may have limited the ability to fully evaluate the additive osteoinductive effects of rhBMP-2. To better assess its therapeutic potential, future studies should consider segmental diaphyseal long bone defect models, where endogenous healing is less robust and the need for osteoinductive support is greater [53, 58]. Finally, although the rhBMP-2 doses used in this study showed biological activity, further dose optimization is necessary to balance efficacy, safety, and cost-effectiveness. Identifying the minimum effective dose while maintaining reliable bone formation will be essential for future clinical translation [59–61].

Despite these limitations, this study provides valuable preclinical insights into the use of MCaP as a carrier for rhBMP-2 in bone regeneration. Through systematic comparison of multiple rhBMP-2 doses in a standardized trabecular defect model, it offers meaningful data on dose–response relationships, carrier efficacy, and early healing dynamics. These findings support the potential of MCaP as a controllable delivery platform for BMP-2 and contribute to the broader understanding of biologic strategies for fracture healing.

## Conclusions

In conclusion, this study demonstrated that a MCaP carrier alone effectively supports bone regeneration in a critical-sized metaphyseal core defect model, confirming its strong osteoconductive capacity. When combined with rhBMP-2, earlier bone healing and faster graft resorption were observed, particularly at higher doses, based on qualitative histological findings.

Additionally, the minimal inflammatory response observed across all groups supports the biocompatibility and safety of this material for potential clinical use. The osteoinductive effect of rhBMP-2 may offer greater benefits in more challenging bone-healing environments, such as diaphyseal or segmental bone defects. Further long-term studies are needed to validate its sustained efficacy and clinical relevance.

## Data Availability

The datasets used and/or analyzed during the current study are available from the corresponding author upon reasonable request.

## Abbreviations

RhBMP-2: Recombinant human bone morphogenetic protein-2
MCaP: Moldable calcium phosphate
HA: Hydroxyapatite
β-TCP: β-Tricalcium phosphate
AP: Anteroposterior
μCT: Microcomputed tomography
PMMA: Polymethyl methacrylate
H&E: Hematoxylin and eosin
ROIs: Regions of interest
BIRI: Bone in the region of interest
SD: Standard deviations
ANOVA: Analysis of variance

## References

[1] Migliorini F, La Padula G, Torsiello E, Spiezia F, Oliva F, Maffulli N. Strategies for large bone defect reconstruction after trauma, infections or tumour excision: a comprehensive review of the literature. Eur J Med Res. 2021;26:118. 34600573 10.1186/s40001-021-00593-9 PMC8487570

[2] Park JS, Kim HJ, Park SJ, Kang DH, Lee CS. A comprehensive review of risk factors and prevention strategies: how to minimize mechanical complications in corrective surgery for adult spinal deformity. Asian Spine J. 2025. 10.31616/asj.2024.0505. Epub ahead of print. PMID: 40033732. 10.31616/asj.2024.0505 PMC12242250 40033732

[3] Kim YH, Kim KW, Rhyu KW, Park JB, Shin JH, Kim YY, Lee JS, Ahn JH, Ryu JH, Park HY, Kim SI. Bone fusion materials: past, present, and future. Asian Spine J. 2025. 10.31616/asj.2024.0520. Epub ahead of print. PMID: 39905833. 10.31616/asj.2024.0520 PMC12242259 39905833

[4] Oryan A, Alidadi S, Moshiri A, Maffulli N. Bone regenerative medicine: classic options, novel strategies, and future directions. J Orthop Surg Res. 2014;9:18. 24628910 10.1186/1749-799X-9-18 PMC3995444

[5] Khanna A, Gougoulias N, Maffulli N. Intermittent pneumatic compression in fracture and soft-tissue injuries healing. Br Med Bull. 2008;88:147–56. 18596049 10.1093/bmb/ldn024

[6] Martinez de Albornoz P, Khanna A, Longo UG, Forriol F, Maffulli N. The evidence of low-intensity pulsed ultrasound for in vitro, animal and human fracture healing. Br Med Bull. 2011;100:39–57. 21429948 10.1093/bmb/ldr006

[7] Agarwal R, Williams K, Umscheid CA, Welch WC. Osteoinductive bone graft substitutes for lumbar fusion: a systematic review. J Neurosurg Spine. 2009;11:729–40. 19951027 10.3171/2009.6.SPINE08669

[8] Kim NH, Jung SK, Lee J, Chang PS, Kang SH. Modulation of osteogenic differentiation by Escherichia coli-derived recombinant bone morphogenetic protein-2. AMB Express. 2022;12:106. 35947236 10.1186/s13568-022-01443-5 PMC9365917

[9] Keum BR, Kim HJ, Kim GH, Chang DG. Osteobiologies for spinal fusion from biological mechanisms to clinical applications: a narrative review. Int J Mol Sci. 2023;24:17365. 10.3390/ijms242417365 PMC10743675 38139194

[10] Khan WS, Rayan F, Dhinsa BS, Marsh D. An osteoconductive, osteoinductive, and osteogenic tissue-engineered product for trauma and orthopaedic surgery: how far are we? Stem Cells Int. 2012;2012: 236231. 25098363 10.1155/2012/236231 PMC3205731

[11] Oliveira ÉR, Nie L, Podstawczyk D, Allahbakhsh A, Ratnayake J, Brasil DL, Shavandi A. Advances in growth factor delivery for bone tissue engineering. Int J Mol Sci. 2021;22:903. 10.3390/ijms22020903 PMC7831065 33477502

[12] Geiger M, Li RH, Friess W. Collagen sponges for bone regeneration with rhBMP-2. Adv Drug Deliv Rev. 2003;55:1613–29. 14623404 10.1016/j.addr.2003.08.010

[13] Govender S, Csimma C, Genant HK, Valentin-Opran A, Amit Y, Arbel R, et al. Recombinant human bone morphogenetic protein-2 for treatment of open tibial fractures: a prospective, controlled, randomized study of four hundred and fifty patients. J Bone Joint Surg Am. 2002;84:2123–34. 12473698 10.2106/00004623-200212000-00001

[14] Boyne PJ, Marx RE, Nevins M, Triplett G, Lazaro E, Lilly LC, et al. A feasibility study evaluating rhBMP-2/absorbable collagen sponge for maxillary sinus floor augmentation. Int J Periodontics Restorative Dent. 1997;17:11–25. 10332250

[15] McKay WF, Peckham SM, Badura JM. A comprehensive clinical review of recombinant human bone morphogenetic protein-2 (INFUSE Bone Graft). Int Orthop. 2007;31:729–34. 17639384 10.1007/s00264-007-0418-6 PMC2266665

[16] Li RH, Bouxsein ML, Blake CA, D’Augusta D, Kim H, Li XJ, et al. rhBMP-2 injected in a calcium phosphate paste (alpha-BSM) accelerates healing in the rabbit ulnar osteotomy model. J Orthop Res. 2003;21:997–1004. 14554211 10.1016/S0736-0266(03)00082-2

[17] Seeherman H, Li R, Bouxsein M, Kim H, Li XJ, Smith-Adaline EA, et al. rhBMP-2/calcium phosphate matrix accelerates osteotomy-site healing in a nonhuman primate model at multiple treatment times and concentrations. J Bone Joint Surg Am. 2006;88:144–60. 16391260 10.2106/JBJS.D.02453

[18] Seeherman HJ, Li XJ, Smith E, Parkington J, Li R, Wozney JM. Intraosseous injection of rhBMP-2/calcium phosphate matrix improves bone structure and strength in the proximal aspect of the femur in chronic ovariectomized nonhuman primates. J Bone Joint Surg Am. 2013;95:36–47. 23283371 10.2106/JBJS.K.00668

[19] Bae HW, Patel VV, Sardar ZM, Badura JM, Pradhan BB, Seim HB 3rd, et al. Transient Local Bone Remodeling Effects of rhBMP-2 in an Ovine Interbody Spine Fusion Model. J Bone Joint Surg Am. 2016;98:2061–70. 28002369 10.2106/JBJS.16.00345

[20] Milovancev M, Muir P, Manley PA, Seeherman HJ, Schaefer S. Clinical application of recombinant human bone morphogenetic protein-2 in 4 dogs. Vet Surg. 2007;36:132–40. 17335420 10.1111/j.1532-950X.2007.00245.x

[21] Bai B, Yin Z, Xu Q, Lew M, Chen Y, Ye J, et al. Histological changes of an injectable rhBMP-2/calcium phosphate cement in vertebroplasty of rhesus monkey. Spine (Phila Pa 1976). 2009;34:1887–92. 19680096 10.1097/BRS.0b013e3181b0e579

[22] Lim HK, Kwon IJ, On SW, Hong SJ, Yang BE, Kim SM, Lee JH, Byun SH. Enhanced bone regeneration in variable-type biphasic ceramic phosphate scaffolds using rhBMP-2. Int J Mol Sci. 2021;22:11485. 10.3390/ijms222111485 PMC8583890 34768914

[23] Lyon T, Scheele W, Bhandari M, Koval KJ, Sanchez EG, Christensen J, et al. Efficacy and safety of recombinant human bone morphogenetic protein-2/calcium phosphate matrix for closed tibial diaphyseal fracture: a double-blind, randomized, controlled phase-II/III trial. J Bone Joint Surg Am. 2013;95:2088–96. 24306695 10.2106/JBJS.L.01545

[24] Schmidlin PR, Nicholls F, Kruse A, Zwahlen RA, Weber FE. Evaluation of moldable, in situ hardening calcium phosphate bone graft substitutes. Clin Oral Implants Res. 2013;24:149–57. 22092691 10.1111/j.1600-0501.2011.02315.x

[25] Tateiwa D, Nakagawa S, Tsukazaki H, Okada R, Kodama J, Kushioka J, et al. A novel BMP-2-loaded hydroxyapatite/beta-tricalcium phosphate microsphere/hydrogel composite for bone regeneration. Sci Rep. 2021;11:16924. 34413442 10.1038/s41598-021-96484-4 PMC8376985

[26] Nakagawa S, Okada R, Kushioka J, Kodama J, Tsukazaki H, Bal Z, et al. Effects of rhBMP-2-loaded hydroxyapatite granules/beta-tricalcium phosphate hydrogel (HA/β-TCP/hydrogel) composite on a rat model of caudal intervertebral fusion. Sci Rep. 2022;12:7906. 35550600 10.1038/s41598-022-12082-y PMC9098867

[27] Kitahara T, Tateiwa D, Hirai H, Ikuta M, Furuichi T, Bun M, et al. rhBMP-2-loaded hydroxyapatite/beta-tricalcium phosphate microsphere/hydrogel composite promotes bone regeneration in a novel rat femoral nonunion model. Front Bioeng Biotechnol. 2024;12:1461260. 39434714 10.3389/fbioe.2024.1461260 PMC11492530

[28] Migliorini F, Cocconi F, Vecchio G, Schäefer L, Koettnitz J, Maffulli N. Pharmacological agents for bone fracture healing: talking points from recent clinical trials. Expert Opin Investig Drugs. 2023;32:855–65. 37740660 10.1080/13543784.2023.2263352

[29] Walsh WR, Oliver RA, Christou C, Lovric V, Walsh ER, Prado GR, et al. Critical Size Bone Defect Healing Using Collagen-Calcium Phosphate Bone Graft Materials. PLoS ONE. 2017;12: e0168883. 28045946 10.1371/journal.pone.0168883 PMC5207671

[30] Walsh WR, Chapman-Sheath PJ, Cain S, Debes J, Bruce WJ, Svehla MJ, et al. A resorbable porous ceramic composite bone graft substitute in a rabbit metaphyseal defect model. J Orthop Res. 2003;21:655–61. 12798065 10.1016/S0736-0266(03)00012-3

[31] Walsh WR, Vizesi F, Michael D, Auld J, Langdown A, Oliver R, et al. Beta-TCP bone graft substitutes in a bilateral rabbit tibial defect model. Biomaterials. 2008;29:266–71. 18029011 10.1016/j.biomaterials.2007.09.035

[32] Nakarai H, Kazarian GS, Lovecchio FC, Kim HJ. Hounsfield units and vertebral bone quality score for predicting mechanical complications after adult spinal deformity surgery: a systematic review and meta-analysis. Asian Spine J. 2024;18:719–30. 39434231 10.31616/asj.2023.0402 PMC11538826

[33] Conway JC, Oliver RA, Wang T, Wills DJ, Herbert J, Buckland T, et al. The efficacy of a nanosynthetic bone graft substitute as a bone graft extender in rabbit posterolateral fusion. Spine J. 2021;21:1925–37. 34033931 10.1016/j.spinee.2021.05.017

[34] van Dijk LA, Barrère-de Groot F, Rosenberg A, Pelletier M, Christou C, de Bruijn JD, et al. MagnetOs, Vitoss, and Novabone in a Multi-endpoint Study of Posterolateral Fusion: A True Fusion or Not? Clin Spine Surg. 2020;33:E276–87. 31977334 10.1097/BSD.0000000000000920 PMC7337107

[35] Crowley JD, Oliver RA, Dan MJ, Wills DJ, Rawlinson JW, Crasto RA, et al. Single level posterolateral lumbar fusion in a New Zealand White rabbit (Oryctolagus cuniculus) model: Surgical anatomy, operative technique, autograft fusion rates, and perioperative care. JOR Spine. 2021;4: e1135. 33778408 10.1002/jsp2.1135 PMC7984023

[36] Malhan D, Muelke M, Rosch S, Schaefer AB, Merboth F, Weisweiler D, et al. An Optimized Approach to Perform Bone Histomorphometry. Front Endocrinol (Lausanne). 2018;9:666. 30519215 10.3389/fendo.2018.00666 PMC6259258

[37] Szymanski L, Golaszewska K, Wiatrowska A, Dropik M, Szymanski P, Gromadka B, et al. ISO 10993 biological evaluation of novel hemostatic powder - 4SEAL®. Biomater Res. 2022;26:12. 35382888 10.1186/s40824-022-00258-6 PMC8981750

[38] Kim RY, Oh JH, Lee BS, Seo YK, Hwang SJ, Kim IS. The effect of dose on rhBMP-2 signaling, delivered via collagen sponge, on osteoclast activation and in vivo bone resorption. Biomaterials. 2014;35:1869–81. 24321706 10.1016/j.biomaterials.2013.11.029

[39] Jeong J, Kim JH, Shim JH, Hwang NS, Heo CY. Bioactive calcium phosphate materials and applications in bone regeneration. Biomater Res. 2019;23:4. 30675377 10.1186/s40824-018-0149-3 PMC6332599

[40] LeGeros RZ. Properties of osteoconductive biomaterials: calcium phosphates. Clin Orthop Relat Res. 2002;395:81–98. 10.1097/00003086-200202000-00009 11937868

[41] Todd EA, Mirsky NA, Silva BLG, Shinde AR, Arakelians ARL, Nayak VV, Marcantonio RAC, Gupta N, Witek L, Coelho PG. Functional scaffolds for bone tissue regeneration: a comprehensive review of materials, methods, and future directions. J Funct Biomater. 2024;15:280. 10.3390/jfb15100280 PMC11509029 39452579

[42] Arias-Betancur A, Badilla-Wenzel N, Astete-Sanhueza Á, Farfán-Beltrán N, Dias FJ. Carrier systems for bone morphogenetic proteins: An overview of biomaterials used for dentoalveolar and maxillofacial bone regeneration. Jpn Dent Sci Rev. 2022;58:316–27. 36281233 10.1016/j.jdsr.2022.10.001 PMC9587372

[43] El Bialy I, Jiskoot W, Reza NM. Formulation, Delivery and Stability of Bone Morphogenetic Proteins for Effective Bone Regeneration. Pharm Res. 2017;34:1152–70. 28342056 10.1007/s11095-017-2147-x PMC5418324

[44] James AW, LaChaud G, Shen J, Asatrian G, Nguyen V, Zhang X, et al. A Review of the Clinical Side Effects of Bone Morphogenetic Protein-2. Tissue Eng Part B Rev. 2016;22:284–97. 26857241 10.1089/ten.teb.2015.0357 PMC4964756

[45] Huang X, Lou Y, Duan Y, Liu H, Tian J, Shen Y, et al. Biomaterial scaffolds in maxillofacial bone tissue engineering: A review of recent advances. Bioact Mater. 2024;33:129–56. 38024227 10.1016/j.bioactmat.2023.10.031 PMC10665588

[46] Szwed-Georgiou A, Płociński P, Kupikowska-Stobba B, Urbaniak MM, Rusek-Wala P, Szustakiewicz K, et al. Bioactive Materials for Bone Regeneration: Biomolecules and Delivery Systems. ACS Biomater Sci Eng. 2023;9:5222–54. 37585562 10.1021/acsbiomaterials.3c00609 PMC10498424

[47] Garg P, Mazur MM, Buck AC, Wandtke ME, Liu J, Ebraheim NA. Prospective Review of Mesenchymal Stem Cells Differentiation into Osteoblasts. Orthop Surg. 2017;9:13–9. 28276640 10.1111/os.12304 PMC6584428

[48] Boerckel JD, Kolambkar YM, Dupont KM, Uhrig BA, Phelps EA, Stevens HY, et al. Effects of protein dose and delivery system on BMP-mediated bone regeneration. Biomaterials. 2011;32:5241–51. 21507479 10.1016/j.biomaterials.2011.03.063 PMC3129848

[49] Inoue S, Takito J, Nakamura M. Site-specific fracture healing: comparison between diaphysis and metaphysis in the mouse long bone. Int J Mol Sci. 2021;22:9299. 10.3390/ijms22179299 PMC8430651 34502206

[50] Halloran D, Durbano HW, Nohe A. Bone morphogenetic protein-2 in development and bone homeostasis. J Dev Biol. 2020;8:19. 10.3390/jdb8030019 PMC7557435 32933207

[51] Liu J, Liu TT, Zhang HC, Li C, Wei W, Chao AJ. Effects of Jintiange on the healing of osteoporotic fractures in aged rats. J Orthop Surg Res. 2024;19:828. 39696674 10.1186/s13018-024-05351-1 PMC11657282

[52] Wang W, Yeung KWK. Bone grafts and biomaterials substitutes for bone defect repair: A review. Bioact Mater. 2017;2:224–47. 29744432 10.1016/j.bioactmat.2017.05.007 PMC5935655

[53] Panos JA, Coenen MJ, Nagelli CV, McGlinch EB, Atasoy-Zeybek A, Lopez De Padilla C, et al. Segmental defect healing in the presence or absence of recombinant human BMP2: Novel insights from a rat model. J Orthop Res. 2023;41:1934–44. 10.1002/jor.25530 PMC10440238 36850029

[54] Jones AL, Bucholz RW, Bosse MJ, Mirza SK, Lyon TR, Webb LX, et al. Recombinant human BMP-2 and allograft compared with autogenous bone graft for reconstruction of diaphyseal tibial fractures with cortical defects. A randomized, controlled trial. J Bone Joint Surg Am. 2006;88:1431–41. 16818967 10.2106/JBJS.E.00381

[55] Huang RL, Yuan Y, Tu J, Zou GM, Li Q. Exaggerated inflammatory environment decreases BMP-2/ACS-induced ectopic bone mass in a rat model: implications for clinical use of BMP-2. Osteoarthritis Cartilage. 2014;22:1186–96. 24981632 10.1016/j.joca.2014.06.017

[56] Cheng L, Wang T, Zhu J, Cai P. Osteoinduction of Calcium Phosphate Ceramics in Four Kinds of Animals for 1 Year: Dog, Rabbit, Rat, and Mouse. Transplant Proc. 2016;48:1309–14. 27320611 10.1016/j.transproceed.2015.09.065

[57] Li Y, Sun Y, Ma K, Wang S, Wang Z, Huang L. Functional mechanism and clinical implications of LINC00339 in delayed fracture healing. J Orthop Surg Res. 2024;19:511. 39192334 10.1186/s13018-024-04998-0 PMC11348643

[58] Stokovic N, Ivanjko N, Maticic D, Luyten FP, Vukicevic S. Bone morphogenetic proteins, carriers, and animal models in the development of novel bone regenerative therapies. Materials (Basel). 2021;14:3513. 10.3390/ma14133513 PMC8269575 34202501

[59] Lytle EJ, Lawless MH, Paik G, Tong D, Soo TM. The minimally effective dose of bone morphogenetic protein in posterior lumbar interbody fusion: a systematic review and meta-analysis. Spine J. 2020;20:1286–304. 32339767 10.1016/j.spinee.2020.04.012

[60] Lee HY, An SB, Hwang SY, Hwang GY, Lee HL, Park HJ, et al. Synergistic enhancement of spinal fusion in preclinical models using low-dose rhBMP-2 and stromal vascular fraction in an injectable hydrogel composite. Mater Today Bio. 2025;30: 101379. 39759847 10.1016/j.mtbio.2024.101379 PMC11699625

[61] Ji X, Zhao D, Xin Z, Feng H, Huang Z. The predictive value of stress-induced hyperglycemia parameters for delayed healing after tibial fracture post-surgery. J Orthop Surg Res. 2024;19:666. 39415173 10.1186/s13018-024-05138-4 PMC11484393

